# Supplementary figures and images for: Spatial approach for diagnosis of yield-limiting nutrients in smallholder agroecosystem landscape using population-based farm survey data
Source: PLoS One. 2022 Feb 2;17(2):e0262754. doi: 10.1371/journal.pone.0262754 (PMC8809601; doi:10.1371/journal.pone.0262754)

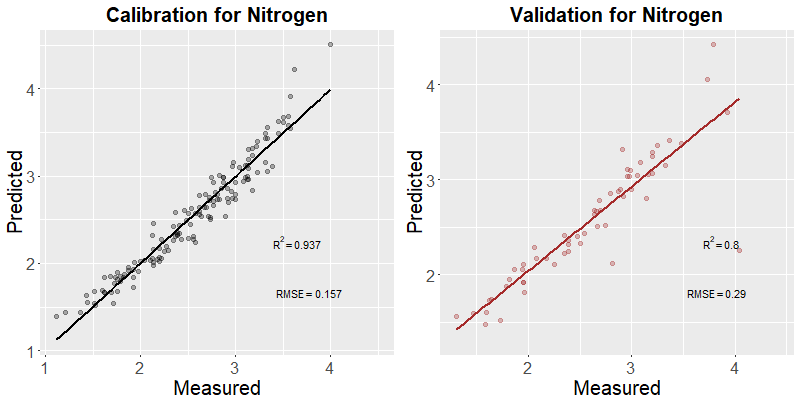

Supplement: S1 Fig — (DOCX) [file pone.0262754.s001.docx]

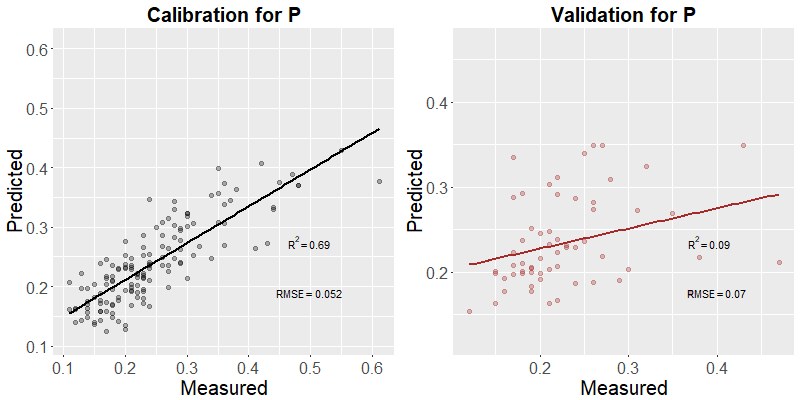

Supplement: S2 Fig — (DOCX) [file pone.0262754.s002.docx]

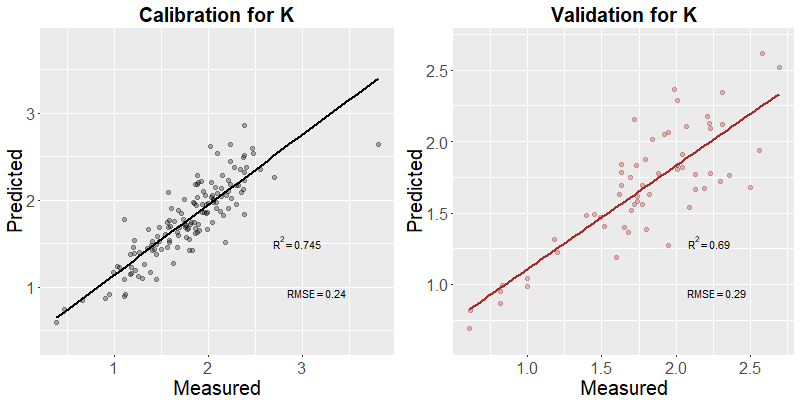

Supplement: S3 Fig — (DOCX) [file pone.0262754.s003.docx]

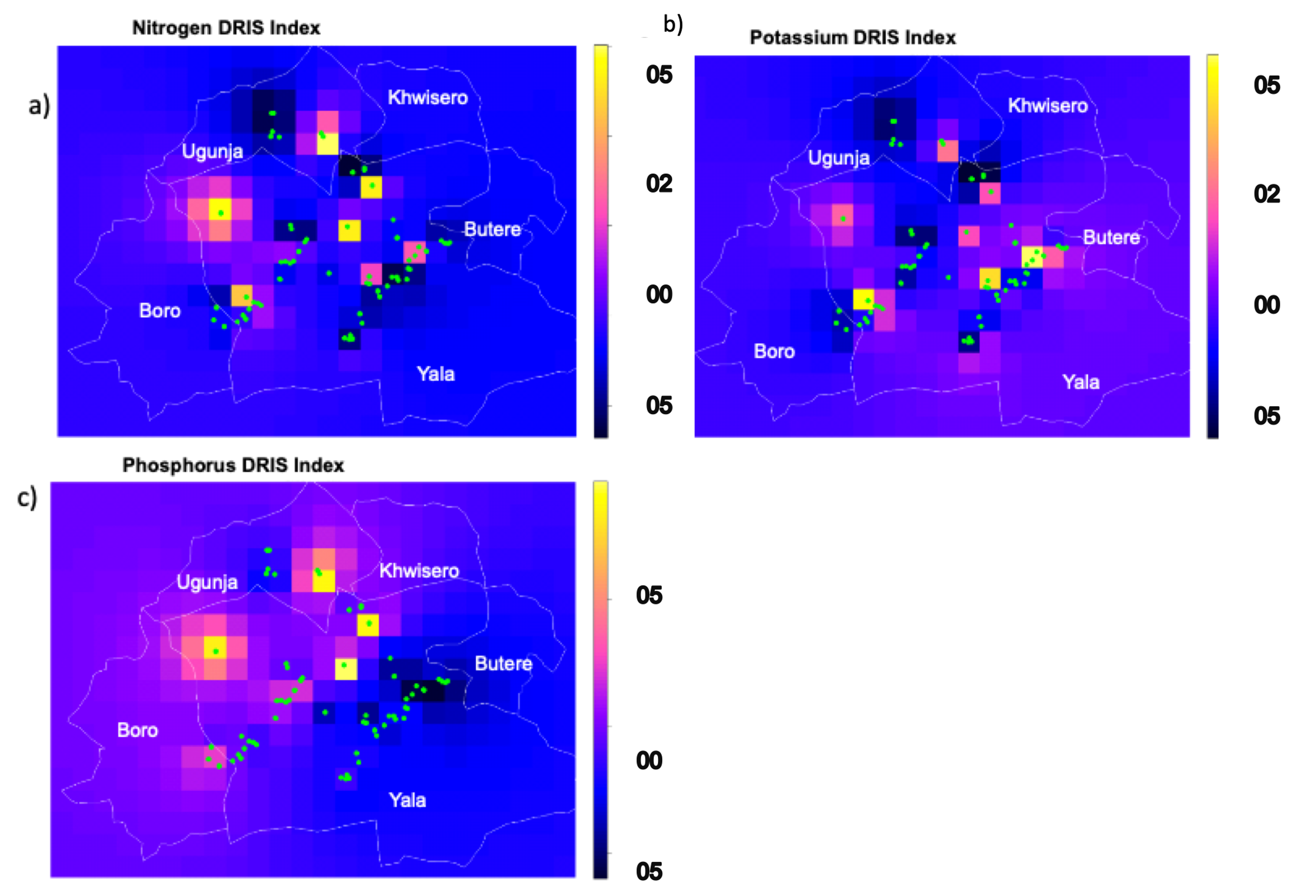

Supplement: S4 Fig — The green points represent the maize fields. The maps were interpolated using inverse distance averages. (DOCX) [file pone.0262754.s004.docx]
