# Supplementary material for: Spatial approach for diagnosis of yield-limiting nutrients in smallholder agroecosystem landscape using population-based farm survey data
Source: PLoS One. 2022 Feb 2;17(2):e0262754. doi: 10.1371/journal.pone.0262754 (PMC8809601; doi:10.1371/journal.pone.0262754)
Supplement: Attachment — Submitted filename: PLOS one Rebuttal- Ichami et al .docx [file pone.0262754.s005.docx]

**Manuscript**

**PONE-D-20-17628
PLOS ONE**

Dear Dr. Elisabeth Bui,

Thank you for giving us the opportunity to re-submit a revised draft of the manuscript; Spatially explicit approach for diagnosis of yield-limiting nutrients in smallholder agroecosystem using population-based farm survey data.

We appreciate the time and effort that you and the two reviewers dedicated to providing feedback on our manuscript and are grateful for the insightful comments and valuable improvements to our manuscript. We have taken time taken to re-read the it and address all the comments. In particular, the details of each comments are provided the following section below:

Please note the reviewer’s comment are in black fonts and our reaction are blue in color.

Thank you

Kind regards

Stephen Ichami

Journal Requirements:

When submitting your revision, we need you to address these additional requirements.

1. Please ensure that your manuscript meets PLOS ONE's style requirements, including those for file naming. The PLOS ONE style templates can be found at

https://journals.plos.org/plosone/s/file?id=wjVg/PLOSOne_formatting_sample_main_body.pdf and

<https://journals.plos.org/plosone/s/file?id=ba62/PLOSOne_formatting_sample_title_authors_affiliations.pdf>

We have checked the manuscript and ensured that it conforms to authors guidelines provided for PLOS one journal.

2. In your Methods section, please provide additional location information, including geographic coordinates for the data set if available.

We have added a map with the coordinated of the sampled maize fields in the supplementary material S1 Table 1.

3. In your Methods section, please provide additional information regarding the permits you obtained for the work. Please ensure you have included the full name of the authority that approved the field site access and, if no permits were required, a brief statement explaining why.

Additional information has been provided in the methodology section line 182 in the revised version.

4. We note that [Figure(s) 1] in your submission contain [map/satellite] images which may be copyrighted. All PLOS content is published under the Creative Commons Attribution License (CC BY 4.0), which means that the manuscript, images, and Supporting Information files will be freely available online, and any third party is permitted to access, download, copy, distribute, and use these materials in any way, even commercially, with proper attribution. For these reasons, we cannot publish previously copyrighted maps or satellite images created using proprietary data, such as Google software (Google Maps, Street View, and Earth). For more information, see our copyright guidelines: http://journals.plos.org/plosone/s/licenses-and-copyright.

We require you to either (1) present written permission from the copyright holder to publish these figures specifically under the CC BY 4.0 license, or (2) remove the figures from your submission:

1. You may seek permission from the original copyright holder of Figure(s) [1] to publish the content specifically under the CC BY 4.0 license.

We recommend that you contact the original copyright holder with the Content Permission Form (http://journals.plos.org/plosone/s/file?id=7c09/content-permission-form.pdf) and the following text:

“I request permission for the open-access journal PLOS ONE to publish XXX under the Creative Commons Attribution License (CCAL) CC BY 4.0 (http://creativecommons.org/licenses/by/4.0/). Please be aware that this license allows unrestricted use and distribution, even commercially, by third parties. Please reply and provide explicit written permission to publish XXX under a CC BY license and complete the attached form.”

Please upload the completed Content Permission Form or other proof of granted permissions as an "Other" file with your submission.

In the figure caption of the copyrighted figure, please include the following text: “Reprinted from [ref] under a CC BY license, with permission from [name of publisher], original copyright [original copyright year].”

2. If you are unable to obtain permission from the original copyright holder to publish these figures under the CC BY 4.0 license or if the copyright holder’s requirements are incompatible with the CC BY 4.0 license, please either i) remove the figure or ii) supply a replacement figure that complies with the CC BY 4.0 license. Please check copyright information on all replacement figures and update the figure caption with source information. If applicable, please specify in the figure caption text when a figure is similar but not identical to the original image and is therefore for illustrative purposes only.

The following resources for replacing copyrighted map figures may be helpful:

USGS National Map Viewer (public domain): http://viewer.nationalmap.gov/viewer/

The Gateway to Astronaut Photography of Earth (public domain):

http://eol.jsc.nasa.gov/sseop/clickmap/

Maps at the CIA (public domain): https://www.cia.gov/library/publications/the-world-factbook/index.html and <https://www.cia.gov/library/publications/cia-maps> publications/index.html

NASA Earth Observatory (public domain): http://earthobservatory.nasa.gov/

Landsat: http://landsat.visibleearth.nasa.gov/

USGS EROS (Earth Resources Observatory and Science (EROS) Center) (public domain): http://eros.usgs.gov/#

Natural Earth (public domain): <http://www.naturalearthdata.com/>

Figure 1 has been updated. The correct image, that address the issues of copy right as aforementioned has been inserted. Hence, we did not to obtain permission to retain the original image of Fig 1, we see no need of seeking for permission as advised.

5. Our internal editors have looked over your manuscript and determined that it is within the scope of our Call for Papers on Plant Phenomics & Precision Agriculture. This collection of papers is headed by a team of Guest Editors for PLOS ONE. Additional information can be found on our announcement page: https://plos.io/phenomics. If you would like your manuscript to be considered for this collection, please let us know in your cover letter and we will ensure that your paper is treated as if you were responding to this call. If you would prefer to remove your manuscript from collection consideration, please specify this in the cover letter.

If is not past the deadline, then should be considered for this issue related to Plant Phenomics & Precision Agriculture.

6. Please clarify in your Data availability statement whether the database will be made available to other researchers. Please ensure that your database meet PLOS ONE requirements (<https://journals.plos.org/plosone/s/submission-guidelines#loc-methods-software-databases-and-tools>).

Yes, we agree to avail the data will be made available

7. During our internal checks, the in-house editorial staff noted that you conducted research or obtained samples in another country. Please check the relevant national regulations and laws applying to foreign researchers and state whether you obtained the required permits and approvals. Please address this in your ethics statement in both the manuscript and submission information.

Permits and approval were sought form the local authorities and the smallholder farmers, owners of the farm where the samples were collected.

8. We note that you have stated that you will provide repository information for your data at acceptance. Should your manuscript be accepted for publication, we will hold it until you provide the relevant accession numbers or DOIs necessary to access your data. If you wish to make changes to your Data Availability statement, please describe these changes in your cover letter and we will update your Data Availability statement to reflect the information you provide.

The data will be submitted to Harvard Dataverse repository- https://dataverse.harvard.edu Harvard, where a doi will be issued for the data.

Additional Editor Comments (if provided):

The two reviewers have made useful suggestions to improve the manuscript, each focusing on different aspects of the work. Please them into account/address their comments in your revision.


[Note: HTML markup is below. Please do not edit.]

**Reviewers' comments:**

Reviewer's Responses to Questions

**Comments to the Author**

1. Is the manuscript technically sound, and do the data support the conclusions?

The manuscript must describe a technically sound piece of scientific research with data that supports the conclusions. Experiments must have been conducted rigorously, with appropriate controls, replication, and sample sizes. The conclusions must be drawn appropriately based on the data presented.

Reviewer #1: Partly

Reviewer #2: Yes

2. Has the statistical analysis been performed appropriately and rigorously?

Reviewer #1: Yes

Reviewer #2: Yes

3. Have the authors made all data underlying the findings in their manuscript fully available?

The [PLOS Data policy](http://www.plosone.org/static/policies.action#sharing) requires authors to make all data underlying the findings described in their manuscript fully available without restriction, with rare exception (please refer to the Data Availability Statement in the manuscript PDF file). The data should be provided as part of the manuscript or its supporting information, or deposited to a public repository. For example, in addition to summary statistics, the data points behind means, medians and variance measures should be available. If there are restrictions on publicly sharing data—e.g. participant privacy or use of data from a third party—those must be specified.

Reviewer #1: Yes

Reviewer #2: Yes

4. Is the manuscript presented in an intelligible fashion and written in standard English?

PLOS ONE does not copyedit accepted manuscripts, so the language in submitted articles must be clear, correct, and unambiguous. Any typographical or grammatical errors should be corrected at revision, so please note any specific errors here.

Reviewer #1: Yes

Reviewer #2: Yes

5. Review Comments to the Author

Please use the space provided to explain your answers to the questions above. You may also include additional comments for the author, including concerns about dual publication, research ethics, or publication ethics. (Please upload your review as an attachment if it exceeds 20,000 characters)

**Reviewer #1:**

The manuscript exhibits novelty in the use of spatial technology to enhance site specific nutrient management and provides extensive methodology to showcase the approach. However, sampling procedures for soil properties characterization was not standardized for characterizing NPK. This may have influenced the outcome for comparison but the authors were silent on it.

Author response: Thank you for pointing out this. The sampling procedure were standardized for filed data collection of population-based survey data within the Land Degradation Sampling Framework (Vågen *et al*., 2010). However, we agree with the reviewer. In the case where the plots (where measurements are made) size may vary. They may affect the outcome of the conclusion, when the plot sizes vary. However, the based on previous guidelines and literature the sampling plots should be a small as possible, to minimize within variability and increase accuracy of the measure soil variable (Baruck *et al*., 2016). If this is adopted there would be much variation across studies outcomes and would also allow comparison with other studies. The use of infrared spectroscopy is also a strategy for standardization of the approach and minimize variability. Though variation in lab chemical methods across labs used to develop reference data for prediction of soil properties using IR may introduce variability.

The study reports of a weak correlation between plant tissue nutrient concentration and maize gran yield but the explanation provided by authors to back this occurrence was not convincing enough. If grain yield did not depend so much on the nutrients in the plant tissue (due to the weak correlation), what could have accounted for the poor performance of grain yield in the study area? Authors should provide clear explanation to this finding. For eg. some deficiencies in some minor nutrients could inhibit the availability of major nutrients to some plants.

We agree with the reviewer. The weak relations been reported previously by other co-authors and gave similar reasons (Kihara *et al.*, 2017). We also agree that the interaction of nutrients can affect maize yield, and this has been addressed in line 403 – 404 of the current manuscript.

**Introduction**
If possible, authors should provide further knowledge on the uncertainty of using the PFS approach should it be deployed in a smallholder agrosystem since it is the first time that is it being used in such a system. Also indicate whether this approach has ever been used in nutrient management elsewhere aside smallholder agrosystems and its efficiency in such systems.

We agree that this is a potential uncertainty in the application of PFS approach for smallholder farming landscapes. We have added possible area of uncertainty in line 120 – 125. The section has adjusted to address this.

**Materials and Methods**

Authors should provide clarity on how the categorization of 'good' and 'bad' maize fields was done. Was it based on expert/indigenous knowledge or visual interpretation - refer to line 18 as this might affect accuracy of the process. Authors could as well provide a reference to verify the assumption of optimum nutrient concentration at 60 - 75 days of maturity - refer to line 206.

We have adjusted the wording on the sentence. We have also added a reference – field guide developed for identification of problems in tropical maize in line 194 – 198 of the current manuscript.

**Results and Discussion**

Results displayed in Table 3 show that out of the 256 collected soil samples, only 245 were analysed for N. What could have accounted for the non-uniformity in the sample size for the soil properties analysis if samples were collected at the same geographical locations?

Accepted and revised – line 382- 383- The were mainly outliers for N that were expunged from the analysis.

Line 437 - In ranking the limiting nutrients was the 256 observations made for the characterization of NPK used or 256 was used for P and K and 245 used for N? And will the differences in sample size not affect the basis for ranking?

Thank you for pointing this out. Although we agree that this is an important consideration, it is good to note that while calculating the DRIS indices the number of observations in put into consideration. The indices were used in ranking. Thus, even though the difference in sample size may affect the results, the calculation does take this into account. Line 316

Line 554 - Authors report that correlation between maize tissue N and plant biomass was strong when r obtained is 0.22. Already they have reported that all correlations were weak due to low nutrient concentration in the study area - lines 384 to 388, so why will they report that r = 0.22 is strong. This is a contradiction and the figure does not support this argument. Further clarity on the outcome is needed. Maybe better relation than P and K but definitely not strong.

We agree with the reviewer’s assessment on the correlations. Accordingly, throughout the manuscript, we have revised and expunged the word strong relationship. Instead, we have used positive relations. However, it important to note that given the previous results obtained by other authors our results are good compared to those given the heterogeneity observed in these systems.

Authors need to provide clarity on explanation given in lines 569 - 577 as to why N fertilizer with lower portions of P and K be recommended if K is the least limiting nutrient and P the most limiting nutrient as stated. The uncertainties associated with the use of DRIS indices can be reported as well.

We agree with the reviewer that this was an oversight on our part. This has been corrected in line 592

Conclusion
In all, authors should provide strong evidence to support that the deficiencies in NPK were actually limiting plant growth and not any other factor since there was no correlation performed between soil NPK nutrients and maize grain yield. The Cate Nelson analysis showed nutrient responses and does not provide a clear link as to the fact that nutrients were not sufficient for the maize crops. Other factors such as soil type and parent rock material may contribute to the responsive nature of the maize to the available soil nutrients. A maize yield distribution map complementing the soil nutrient map may provide further clarity with a bit of literature detailing the soil characteristics of the study area.

We agree with this valuable point that other factors may influence the performance of maize. This was taken into account while sampling thus we only sampled well managed unfertilized plots (line 176) within the study area to minimize variation caused by other factors.

**General comments**

The manuscript is good and publishable and so the authors should be encouraged to make it better to contribute to the knowledge of soil science and geospatial analysis. Minor grammatical errors were detected and must be corrected to make the document more suitable.

We thank the reviewer for the positive comment and agree with the reviewer’s assessment on grammatical errors. In this version we have re-read the manuscript to ensure that we have corrected most grammatical mistakes.

**Reviewer #2:**

Report of PONE-D-20-17628

Ichami et al. “Novel approach for diagnosis of limiting nutrients in smallholder agro-ecosystems using farm survey data”

Ichami et al. outline an interesting approach for diagnosing nutrient deficiency for plant growth combined with recommendations for site/plot specific fertilizer management of smallholder agro-ecosystems in western Kenya. Through what they term population-based farm surveys, guided by subdividing a 100km2 area into hierarchical blocks down to the farm plot level, yield performance and the soil’s nutrient status is assessed by attempting to also taking into account the plot’s geographic location and local surrounding soil variability. The methods used in this study and the aim of using these are well explained, and seem appropriate. Results are described, visualized and assessed appropriately, also in relation to the relevant literature. Although the study was conducted 7 years ago, in 2013, publication still seems relevant. I wonder, however, if the approach was tested for a different crop/region since then.

We thank the reviewer for the positive comment. Yes the approach has been applied in Malawi (Beedy *et al.*, 2015)

The following provides specific comments that should be addressed before publication of the research in PlosOne.

Specific comments:

Keywords; using digital soil mapping as a keyword is a bit misleading, as the research identified ‘localised hot spots’, and did not produce surface maps of soil nutrient distributions across the study area using DSM methods

We agree with the reviewer and we have change this as advised.

**Abstract/Introduction**
It becomes clear that this kind of ‘regional-scale’ assessment is only feasible when combined with rapid and cost-effective soil/plant characterizations utilizing spectroscopic measurement techniques such as MIR, which was used here. This is not clearly stated in the abstract or introduction of the manuscript. Although outlined in the Materials and Methods and further discussed, in my opinion, the rapid/cost-effective assessment is an integral part of the assessment approach and needs to be included/framed upfront.

We have added the suggested content to the manuscript on line 33 – 34 in the abstract and Introduction line 107 to 109

Materials and Methods

L219; also include the processing of plant samples prior to MIR analysis

This been updated, such that the MIR analysis are clearly indicated in line 233 - 235

L234; please clearly state for the reader that the selection of sub-samples to be sent off for conventional lab analysis was based on the MIR spectra, to gain a representative sub-sample of the variation contained in the dataset to establish predictive models; please also state which algorithm was used for sub-sampling (e.g. cLHS, Kennard-Stone…)

This been updated, to include the algorithm used for sub-sampling in line 260 – 263

L244 onwards; were soil/plant attribute data transformed prior to the generation of regional calibration models. This is not clear.

This been updated, and clarified in line – 277 – 278

Results
Figure 9, the nutrient deficiency ‘hot spot maps’ would benefit from also showing a map of soil types of the study region (if available), to show spatially the distribution of local soil types and how they relate to the localized areas of nutrient deficiency or optimal nutrient status, as some soil types are already inherently depleted of nutrients as discussed in the manuscript (e.g., the Gley soils). Maps of soil type and/or soil attribute distribution can also play a role in identifying representative ‘target populations’ when used as environmental covariates.

We agree with this but the maps important and we recommendation future studies to explore ways in which environmental covariate can be incorporated in PFS as this would make it more robust approach.

In general, the manuscript reads well, however, there are a couple of grammatic errors throughout the manuscript that can be easily rectified through another proof-read. Below I’ve listed a couple of specific situations where a word was missing or where the meaning of a sentence is not clear to the reader.

We appreciate this positive comment from the reviewer.

L34-35, please reword for clarification; e.g. separate between the relationships investigated here, one of these are the relationships between maize yield and soil attributes to establish critical limits where change in attributes are likely to trigger a crop response

This been updated, line 36 -37.

L217; …characterization of soil and plant samples

Line 225 in the current version, accepted and updated

L319; All maps were derived using the ggplot2 R package.

Line 331 in the current version, accepted and updated

L425; Consider changing this sentence to: However, deficient and sufficient populations were separated more clearly for ext. P frequency distributions (Figure 7d).

Accepted and updated changed.

L471; please reword this sentence for clarity

Addressed 483 - 490.

L497-498; please reword this sentence for clarity

Agreed and addressed 509 - 513.

L528; delete the word “limit” after “limiting”

Deleted as advised. Line 542

L534-535; please reword this sentence for clarity

L554; please change the wording “strong correlation” as this is not supported by the data, there is a trend showing a slightly positive relationship between maize tissue N concentration and plant biomass

Agreed and changed now line 569

L566; …, which can be used…

Agreed and delete now line 582

6. PLOS authors have the option to publish the peer review history of their article ([what does this mean?](https://journals.plos.org/plosone/s/editorial-and-peer-review-process#loc-peer-review-history)). If published, this will include your full peer review and any attached files.


If you choose “no”, your identity will remain anonymous but your review may still be made public.


Do you want your identity to be public for this peer review? For information about this choice, including consent withdrawal, please see our [Privacy Policy](https://www.plos.org/privacy-policy).

Reviewer #1: No

Reviewer #2: No

[NOTE: If reviewer comments were submitted as an attachment file, they will be attached to this email and accessible via the submission site. Please log into your account, locate the manuscript record, and check for the action link "View Attachments". If this link does not appear, there are no attachment files.]

While revising your submission, please upload your figure files to the Preflight Analysis and Conversion Engine (PACE) digital diagnostic tool, <https://pacev2.apexcovantage.com/>. PACE helps ensure that figures meet PLOS requirements. To use PACE, you must first register as a user. Registration is free. Then, login and navigate to the UPLOAD tab, where you will find detailed instructions on how to use the tool. If you encounter any issues or have any questions when using PACE, please email PLOS at [figures@plos.org](mailto:figures@plos.org). Please note that Supporting Information files do not need this step.
