# Supplementary material for: Spatial approach for diagnosis of yield-limiting nutrients in smallholder agroecosystem landscape using population-based farm survey data
Source: PLoS One. 2022 Feb 2;17(2):e0262754. doi: 10.1371/journal.pone.0262754 (PMC8809601; doi:10.1371/journal.pone.0262754)
Supplement: Attachment — Submitted filename: PONE-D-21-15324 review.docx [file pone.0262754.s006.docx]

**Reviewer Recommendation and Comments for Manuscript Number PONE-D-21-15324**

Reviewer 3

5. Review Comments to the Author

Please use the space provided to explain your answers to the questions above. You may also include additional comments for the author, including concerns about dual publication, research ethics, or publication ethics. (Please upload your review as an attachment if it exceeds 20,000 characters) (Limit 200 to 20000 Characters)

General comments

The paper is publishable due to the novelty of the approach. In some instances more care needs to be taken to interpret the data presented in figures accurately within text. There are several grammatical errors that can be easily fixed with another proof read. I have noted some of these below as examples, but not all.

**Materials and methods**

Line 177 captured to capture

Line 184 form to from

Line 287 Cate-Nelson (global)

Line 287 cut of to cut-off

Line 311; seems to indicate DRIS indices are calculated form sufficient sub-population only. But lines 457 to 458 indicate DRIS used high and low yielding populations? Not sure if I missed something here?

**Results**

Line 358; listing the twelve variables somewhere would be useful.

Line 382; (table 3 and Figure 5b). If the high N values omitted had low biomass volume this would weaken the relationship of figure 5b.

Line 394. If the figures of 67% and 30% come from equations within figures 5c and 5d respectively then they indicate an increase of 0.67 and 0.30 in P ear-leaf conc (%). i.e. for a 1% increase in leaf P conc yield increased 5.37Mg/ha.

Line 396. I cannot see the two-fold increases in both GY and BV in figures 5 b, c, and e. Weak relationships between tissue nutrient concentration and yield are not unexpected given all the other production constraints at play. Need to be accurate and consistent throughout the document with interpretation of figure 5.

i.e. Line 565 “ The relationship measured between ear-leaf tissue conc and crop response was very tenuous (Fig 5)” versus

Line 577 “strong and significant correlations between N concentration and GY are in line with those of xxx” (the data does not show this).

Line 406; while some biotic production constraints are dealt with in methods, by describing that the paddocks were well managed for weeds and disease and soil type is mentioned there is no mention of abiotic production constraints. Perhaps it would be useful to provide a better summary of climate data in section 2.1 if it is available; including a measure of variability of rainfall and temperature by sites.

Line 419. 67% is a large proportion of the sample. Perhaps “Only” should be removed?

Line 464-468; Extractable P ranked as the most limiting nutrient. Perhaps the long tail of Figure 7c indicates some high P buffering soils. I note line 144 ”majority of soils were Ferrosols”. Also Figure 7d shows that deficient crops (based on tissue testing) tended to come from sites with higher soil P concentration, which might also indicate plant availability of P was lower in some soils.

**Discussion**

Line 577 “strong and significant correlations between N concentration and GY are in line with those of xxx” (the data does not show this).

Line 586. Soil tests and foliar tests can be used independently. Perhaps reword “cannot” to something like… are better used together rather than in isolation.

**Conclusion**

Line 636, Should it be acknowledged that plant (tissue conc.) relationships were weak? Plant relationships, although weak, were used in combination with soil tests to develop soil test values for N, P, K which were then used to define cases of nutrient deficiency. ?

**Figures**

Figure 1.

Line 139 has a different list of sub-regions with data. i.e. Boro is not listed at line 139 but has data pints in figure 1.

Figure 2.

Remove the duplication of “level of” in “Step 3”

Figure 5.

Ensure the data is interpreted correctly throughout the document, as outlined above.

Figure 9.

I found this figure confusing due to the two legends both with colours and the same title.

For 9a line 463 has the range of DRIS for N at -35 to +37, but the legend scale is -30 to +10

For 9c sub-regions are labelled incorrectly in 9c.

For 9d I assume these figures relate to the coloured legend rather than grey scale legend.
